# A Key Marine Diazotroph in a Changing Ocean: The Interacting Effects of Temperature, CO_2_ and Light on the Growth of *Trichodesmium erythraeum* IMS101

**DOI:** 10.1371/journal.pone.0168796

**Published:** 2017-01-12

**Authors:** Tobias G. Boatman, Tracy Lawson, Richard J. Geider

**Affiliations:** School of Biological Sciences, University of Essex, Colchester, United Kingdom; Mount Allison University, CANADA

## Abstract

*Trichodesmium* is a globally important marine diazotroph that accounts for approximately 60 − 80% of marine biological N_2_ fixation and as such plays a key role in marine N and C cycles. We undertook a comprehensive assessment of how the growth rate of *Trichodesmium erythraeum* IMS101 was directly affected by the combined interactions of temperature, *p*CO_2_ and light intensity. Our key findings were: low *p*CO_2_ affected the lower temperature tolerance limit (T_min_) but had no effect on the optimum temperature (T_opt_) at which growth was maximal or the maximum temperature tolerance limit (T_max_); low *p*CO_2_ had a greater effect on the thermal niche width than low-light; the effect of *p*CO_2_ on growth rate was more pronounced at suboptimal temperatures than at supraoptimal temperatures; temperature and light had a stronger effect on the photosynthetic efficiency (*F*_*v*_*/F*_*m*_) than did CO_2_; and at T_opt_, the maximum growth rate increased with increasing CO_2_, but the initial slope of the growth-irradiance curve was not affected by CO_2_. In the context of environmental change, our results suggest that the (i) nutrient replete growth rate of *Trichodesmium* IMS101 would have been severely limited by low *p*CO_2_ at the last glacial maximum (LGM), (ii) future increases in *p*CO_2_ will increase growth rates in areas where temperature ranges between T_min_ to T_opt_, but will have negligible effect at temperatures between T_opt_ and T_max_, (iii) areal increase of warm surface waters (> 18°C) has allowed the geographic range to increase significantly from the LGM to present and that the range will continue to expand to higher latitudes with continued warming, but (iv) continued global warming may exclude *Trichodesmium* spp. from some tropical regions by 2100 where temperature exceeds T_opt_.

## Introduction

The ocean is a major sink for anthropogenic emissions [1], of which the capacity to store CO_2_ is strongly affected by biological processes [2]. As atmospheric CO_2_ increases, the dissolved inorganic carbon (DIC) concentrations in the oceans increases, the pH declines, and the inorganic speciation changes [3]. Such changes are expected to have diverse effects for marine ecosystems [4]. In addition to ocean acidification, climate change will concurrently lead to increases of sea surface temperature (SST) thereby enhancing water stratification and decreasing vertical mixing [5].

One of the most important phytoplankton groups in the open oceans are diazotrophic cyanobacteria, which convert N_2_ gas into ammonia (NH_3_) by nitrogenase activity prior to assimilation of this fixed N into organic matter. Their distributions range from the warm (~ 27°C) tropical waters around the equator (e.g. *Trichodesmium* and *Crocosphaera* spp.), to the colder (~ 5°C) waters of the Baltic Sea (e.g. *Aphanizomenon* sp. and *Nodularia* spp.) [6,7]. Diazotrophs found at cold temperatures possess specialised cell compartments (heterocysts) where N_2_ fixation occurs [8]. Conversely, all equatorial diazotrophic cyanobacteria are non-heterocystous. The non-heterocystous filamentous, diazotroph *Trichodesmium* spp. are estimated to contribute significantly to global productivity and to biogeochemical cycles [9], and are considered to be the dominant equatorial diazotrophs [10–12], representing up to 50% of new nitrogen in some regions [13,14], and contributing between 80 and 110 Tg of fixed N_2_ to the open ocean ecosystems per year [11], although there is evidence for significant contributions to N_2_ fixation by unicellular cyanobacteria [15].

*Trichodesmium* spp. often form extensive surface blooms where cells are exposed to high temperatures, high light intensities, fluctuating nutrient regimes and water column mixing [16,17], all of which can contribute to the high degree of spatial and temporal variability in trichome densities [13,18]. In the eastern Atlantic Ocean, *Trichodesmium* spp. are most abundant from 0 to 15°N, with complete absence south of 30°S [19]. *Trichodesmium* is commonly observed from the upper few meters of the water column where the surface light intensity is ~ 2500 μmol photons m^-2^ s^-1^, to ~ 100 meters depth where the 0.5 to 1% light level is reached (~ 12.5–25 μmol photons m^-2^ s^-1^) and temperatures are between 21 and 23°C [20,21].

Knowledge of how the filamentous diazotroph *Trichodesmium* responds to temperature, light and *p*CO_2_ is critically important to understand the potential implications of global warming and ocean acidification on future global biogeochemical cycles, food web dynamics, and overall productivity of the open oceans [22].

The effect of temperature on growth rate can be characterised by a thermal tolerance curve (μ-T curve); where an organism’s growth is constrained between a maximum (T_max_) and minimum (T_min_) temperature limit. The temperature range between T_min_ and T_max_ is defined as the thermal niche, and can vary subject to an organism’s physiological plasticity and evolutionary history [23]. Two features of a growth-temperature curve common to all ectotherms are unimodality and negative skewness [24,25]. Negative skewness describes the sudden sharp decline in fitness above T_opt_, and indicates that when acclimated to T_opt_, growth is more significantly reduced by warming than cooling, a trait of particular relevance given the predicted increase in SST over the coming decades.

The constraint that temperature imposes on the physiology of diazotrophs in general and *Trichodesmium* spp. in particular is still not fully understood, and yet is fundamentally important when trying to predict future productivity and global distributions. Recent research suggests that the minimum temperature limit (T_min_), optimum temperature (T_opt_), and maximum temperature limit (T_max_) for *Trichodesmium erythraeum* growth occurs between 20 − 22°C, 26 − 27°C and 32 − 35°C, respectively; with little variation found between strains or isolates (IMS101, KO4-20, RLI and 2175) [26–28].

Although some research has shown that the growth rate of *Trichodesmium erythraeum* IMS101 is unchanged or decreases with increased CO_2_ concentrations above current ambient levels, the majority of research indicates that an increase in CO_2_ whilst all other factors are kept constant, will result in an increase in growth (Table 1). As is the case for the majority of experiments investigating the effects of ocean acidification [29], most research involving *Trichodesmium* has used a single independent variable (e.g. CO_2_ whilst keeping temperature and light constant or temperature whilst keeping CO_2_ and light constant), of which there are typically 3 to 8 treatments and all cultured for short time periods and often with several undefined growth conditions (Table 1).

**Table 1:** The current literature regarding the effects of varying temperature (°C), CO_2_ (ppm), light intensity (μmol photons m^-2^ s^-1^) and L:D (hr:hr) period on *Trichodesmium erythraeum* IMS101 growth.

Growth Conditions				Acclimation (Generations)	*μ* (d^-1^)	Effect of OA on Growth	Experimental Methods			Ref
Temperature	Light	CO_2_	L:D				Carbon Chemistry	Parameters Defined	Growth Index	
20	100	−	12:12	15	0.04	−	−	−	Chl *a*/C-specific	[26]
21					0.10					
24					0.18					
26					0.25					
30					0.21					
31					0.15					
34					0.07					
24	140	−	14:10	−	0.38	−	−	−	Chl *a*-specific	[30]
26					0.65					
28					0.77					
31					0.64					
25	70	−	8:16	10–40	0.21	−	−	−	Chl a-specific	[31]
			12:12		0.30					
			16:8		0.45					
	350		8:16		0.30					
			12:12		0.29					
			16:8		0.12					
25	150	180	14:10	35	0.26	→	NaOH Additions	TA & TCO_2_	Chl *a*-specific	[32]
		380			0.41					
		550			0.44					
		720			0.45					
		800			0.46					
26	120	380	12:12	> 800	0.26	↑	BubblingGas Mixture	pH & TCO_2_	Cell-specific	[33]
		750			0.37					
25	80–120	250	12:12	5	0.13	↑	BubblingGas Mixture	Headspace *p*CO_2_	Chl *a*/C-specific	[34]
		400			0.16					
		900			0.26					
25	150	150	12:12	5	0.35	→/↓	BubblingGas Mixture	TA & pH	Chl *a*/C-specific	[35]
		370			0.29					
		1000			0.32					
27	90	380^a^	14:10	−	0.26	↓	Bubbling Gas Mixture/NaOH Additions	−	Chl *a*/C-specific	[36]
		750^a^			0.19					
		380^b^			0.46	↓				
		750^b^			0.37					
25	150	180	12:12	7	0.36	↓	Bubbling Gas Mixture	pH & TCO_2_	Chl *a*-specific	[37]
		380			0.34					
		980			0.32					
		1400			0.27					
		180^c^		11	0.34	↓				
		380^c^			0.37					
		980^c^			0.35					
		1400^c^			0.29					
25	80–100	400	12:12	47	0.37	↑	Bubbling Gas Mixture	−	Cell-specific	[38]
		900			0.58					
		400^d^			0.22	↑				
		900^d^			0.31					
25	50	150	12:12	10	0.15	↑	Bubbling Gas Mixture	TA & pH	Chl *a*/C-specific	[39]
		900			0.24					
	200	150			0.38	↑				
		900			0.42					
24	38	Ambient	12:12	7–10	0.12	↑	Bubbling Gas Mixture	pH & TCO_2_	Cell-specific	[40]
	100				0.25					
	220				0.30					
	38	750			0.12					
	100				0.32					
	220				0.38					
26	260^e^	−	12:12	−	0.35	−	−	−	Cell-specific	[41]
	670^e^				0.48					
	260^f^				0.38					
	670^f^				0.49					
25	100	380	12:12	7–10	0.35	↑	Bubbling Gas Mixture	pH & TCO_2_	Cell-specific	[42]
		750			0.39					
29		380			0.36	↑				
		750			0.41					
25	80	400	12:12	10	0.18	↑	Bubbling Gas Mixture	Headspace *p*CO_2_	Chl *a*-specific	[43]
		900			0.32					
31		250			0.26	↑				
		400			0.27					
		900			0.38					

A dash (−) represents no method for controlling the carbon chemistry or an undefined growth condition. Arrows represent an increase (↑), decrease (↓) or negligible (→) effect of ocean acidification (OA) on growth. ImageJ was used to obtain growth rates from figures reported in literature. Note that superscripts in the CO_2_ column indicate the following differences from the standard YBCII culture medium

^a^ = 40 pM Fe´

^b^ = 1250 pM Fe´

^c^ = 100 μM NO_3_^-^

^d^ = 0.5 μM P

^e^ = 20 nM Ni and

^f^ = 100 nM Ni.

To investigate the integrated effects of key physical/chemical variables (temperature, *p*CO_2_ and light) that will be altered by climate change on the growth rate of *Trichodesmium*, we performed a systematic, multivariable experiment, where *Trichodesmium* IMS101 was cultured over long durations (> 9 months), at multiple treatments (*n* = 174) with controlled and defined growth conditions, thus ensuring that balanced growth and complete physiological acclimation was achieved (S1 Table). The aims were to assess the response of *Trichodesmium* IMS101 growth to temperature, CO_2_ and light intensity. We tested the hypotheses that (i) the thermal niche width of *Trichodesmium* IMS101 is reduced (i.e. T_min_ increased and/or T_max_ decreased) under suboptimal light or *p*CO_2_, (ii) the optimal temperature (T_opt_) for growth is unaffected by light or *p*CO_2_, (iii) the maximum photochemical efficiency of PSII (*F*_*v*_*/F*_*m*_) is affected by suboptimal and supraoptimal temperature, light and CO_2_, and (iv) when light is limiting, suboptimal *p*CO_2_ further reduces growth rate.

## Materials and Methods

*Trichodesmium erythraeum* IMS101 was semi-continuously cultured to achieve fully acclimated balanced growth across a range of temperatures (19 − 32°C), light intensities (10–1400 μmol photons m^-2^ s^-1^) and targeted *p*CO_2_ concentrations (180, 380 and 720 ppm).

### Experimental setup

Cultures were grown at low volumes (5 mL) in 12 mL glass test tubes. Test tubes were acid washed and autoclaved prior to culturing, and each dilution was made into a new tube to avoid the build-up of contaminants. Growth rates were quantified from changes in fluorescence (*F*_*o*_) measured daily (between 09:00 to 10:30) on dark-adapted cultures (20 minutes) using a FRRfII Fastact Fluorometer (Chelsea Technologies Group Ltd, UK). The FRRfII parameters were optimised prior to the experiment to ensure a saturating fluorescence curve was achieved for both low (post-dilution) and high (pre-dilution) cell density cultures.

Cultures were kept at the lower section of the exponential growth phase (S1 Fig) and optically thin to avoid nutrient limitation, self-shading and minimise CO_2_ drift [32]. Tubes were gently inverted twice a day to minimise trichomes aggregating at the meniscus. Subject to the temperature and CO_2_, high light cultures were usually diluted every fourth to fifth day, while low light cultures every tenth to twelfth day. When *F*_*o*_ declined at an extreme growth condition (e.g. high temperature), three attempts were made to re-grow that treatment, using culture from the closest growth condition.

### Culture medium and carbonate chemistry

A single batch (25 L) of filter-sterilised (0.25 μm pore) YBCII media [44] was made and stored in acid-washed, autoclaved Duran bottles (no headspace). The inorganic carbon chemistry of each bottle was determined via *CO2SYS* [45]; using a 15 mL sample for TCO_2_ analysis (Shimadzu TOC-V Analyser & ASI-V Autosampler), and a 10 mL sample for pH (Thermo Scientific Orion Ross Ultra pH Electrode EW-05718-75, UK). The pH probes were rinsed and calibrated with fresh (< 2 weeks) artificial seawater buffers (TRIS and AMP) prior to use [46].

Once a culture reached a pre-determined *F*_*o*_ value, it was diluted (0.5 mL culture to 4.5 mL media) with filter-sterilised (0.2 μm pore) YBCII media that had been adjusted to a target pH (and thus target CO_2_) to return the culture to a starting *F*_*o*_ value. To obtain a targeted CO_2_ concentration in the YBCII media used to dilute the semi-continuous cultures, the medium was bubbled with a CO_2_-air mixture to a targeted pH (precision of ± 0.002). Once the media reached the desired CO_2_ concentration (± 1%) it was immediately distributed into the test tubes, already containing the culture (S1 File). Test tubes were sealed via a PTFE lined screw cap and PTFE tape on the test tube threads, ensuring a gas-tight seal and preventing exchange of CO_2_ with the atmosphere. Prior to screwing the cap on, the gaseous headspace was flushed with a filtered (0.2 μm pore) standard gas mixture of a target CO_2_ concentration (BOC Industrial Gases, UK). All carbon chemistry calculations were made in *CO2SYS* [45], using the 1^st^ and 2^nd^ equilibrium constants (K1 and K2) for carbonic acid [47], the dissociation constant for KSO_4_ [48], the boric acid constant (KB) [49], and the total pH scale. The *CO2SYS* program calculates CO_2_ concentrations as μatm; however, as the CO_2_ concentrations reported here are to zero decimal places the equivalent units of parts per million are used (ppm).

Prior to every dilution, 3.5 mL of culture was collected in a 5 mL plastic cryogenic vial (Sigma-Aldrich V5257-250EA) and was used to measure the post-culturing pH. Assuming alkalinity remained constant throughout the entire growth phase [50], a post-culturing CO_2_ was calculated using the post-culturing pH and initial alkalinity.

### Temperature gradient

To measure the effect of temperature on growth, a custom-made water-jacketed aluminium temperature block was used to house test tube cultures. The temperature ranged from 18 to 33°C, and the temperature steps along the gradient were at ~ 0.5°C increments. The temperature (± 0.1°C) of each tube was measured using a temperature probe (Sper Scientific 840038, Arizona USA), and varied < 0.2°C over a diel period for any given culture along the gradient. A clear Perspex sheet was secured beneath of the block to hold the tubes in place and allow illumination from below. Light was provided by four fan-cooled LED strips (The Optoelectronic Manufacturing Corporation Ltd. 3ft T5 Daylight, UK), and was adjusted using neutral density filters, with half of the test tubes illuminated at low light (40 μmol photons m^-2^ s^-1^ ± 0.4), and the remaining tubes at high light (400 μmol photons m^-2^ s^-1^ ± 4), all at a 12:12 L:D cycle. The light was measured for each test tube using a light meter (Li-Cor Li-250A, Nebraska USA), and was checked weekly throughout the experiment. For each of the low and high light treatments, and for each temperature treatment, *Trichodesmium* IMS101 was cultured at three targeted CO_2_ concentrations (180, 380 and 720 ppm), giving a total of 120 treatments.

### Light gradient

To measure the light response of growth a second temperature block was setup and run concurrently. All test tube cultures were maintained at 26°C. Using neutral density filters, a light gradient was setup ranging from 10 to 1400 μmol photons m^-2^ s^-1^, at a 12:12 L:D cycle. The light steps along the gradient were distributed in order to resolve the light-limited and saturated sections of the growth curve. For each light treatment, *Trichodesmium* IMS101 was cultured at three targeted CO_2_ concentrations (180, 380 and 720 ppm), giving a total of 54 treatments.

The measured *p*CO_2_ in the cultures just prior to dilution into fresh medium were between 20% and 30% lower than the target *p*CO_2_ concentrations of 180, 380 and 720 ppm, and were similar across all temperatures and light intensities (Table 2).

**Table 2:** The mean (± S.E.) growth conditions of *Trichodesmium erythraeum* IMS101 cultures for the temperature and light response.

		Temperature response						Light response		
		Low CO_2_		Mid CO_2_		High CO_2_		Low CO_2_	Mid CO_2_	High CO_2_
Variables	Units	LL	HL	LL	HL	LL	HL			
pH	Total	8.506	8.477	8.171	8.175	7.901	7.908	8.524	8.214	7.957
H^+^	nM	3.1(±0.02)	3.3(±0.032)	6.7(±0.044)	6.7(±0.041)	12.6(±0.074)	12.3(±0.056)	3.0(±0.016)	6.1(±0.033)	11.0(±0.074)
A_T_	μM	3275(±22)	2993(±25)	2723(±21)	2796(±16)	2549(±17)	2626(±11)	3004(±6)	2963(±9)	2936(±93)
TCO_2_	μM	2387(±23)	2187(±18)	2242(±19)	2298(±14)	2264(±15)	2332(±10)	2146(±5)	2410(±7)	2583(±14)
HCO_3_^-^	μM	1765(±23)	1639(±15)	1922(±17)	1962(±12)	2067(±13)	2127(±9)	1562(±5)	2031(±5)	2330(±12)
CO_3_^2-^	μM	618(±5)	544(±9)	313(±4)	326(±4)	178(±3)	186(±2)	580(±2)	370(±2)	235(±2)
CO_2_	μM	3.89(±0.09)	3.85(±0.08)	9.14(±0.11)	9.17(± 0.10)	18.41(±0.18)	18.64(±0.15)	3.25(±0.03)	8.62(±0.05)	17.86(±0.06)
*p*CO_2_	ppm	139(±2)	138(±2)	325(±3)	330(±2)	649(±3)	658(±2)	118(±1)	312(±2)	647(±2)
Chl *a*	μg L^-1^	15.9(±1.1)	16.2(±1.0)	23.0(±1.7)	19.6(±1.5)	25.4(±1.8)	20.6(±1.7)	21.8(±1.3)	28.8(±1.6)	36.3(±2.1)
*n*		46	63	74	78	83	98	107	124	102

For the temperature response, the growth conditions per CO_2_ and light treatment is the average of all temperature treatments. For the light response, the growth conditions per CO_2_ treatment is the average of all light treatments. The total inorganic carbon (TCO_2_), bicarbonate (HCO_3_^-^), carbonate (CO_3_^2-^) and *p*CO_2_ was calculated via *CO2SYS* using the pH and A_T_ concentration. Temperature response growth conditions; low (180 ppm), mid (380 ppm) and high (720 ppm) CO_2_, 40 μmol photons m^-2^ s^-1^ (LL) and 400 μmol photons m^-2^ s^-1^ (HL), ranging between 18–31°C. Light response growth conditions; low (180 ppm), mid (380 ppm) and high (720 ppm) CO_2_, 26°C, ranging between 20–1400 μmol photons m^-2^ s^-1^)

### Chlorophyll fluorescence

In addition to the minimum fluorescence (*F*_*o*_), the FastPro software (Chelsea Technologies Group Ltd, UK) generated dark-adapted values of maximum fluorescence (*F*_*m*_). The maximum photochemical efficiency of PSII in the dark-adapted state (*F*_*v*_*/F*_*m*_) was calculated using the following:

$$\frac{F_{v}}{F_{m}}=\left(\frac{F_{m}-F_{o}}{F_{m}}\right)$$ (1)

### Data processing to obtain balanced growth rates

Acclimation took ~ 12 to 16 weeks with up to 20 dilutions required to allow cultures to achieve balanced growth at the extreme temperature and light limits. For each dilution, a growth rate was calculated from linear regression of ln(*F*_*o*_) versus time. Balanced growth was assumed to have been achieved once growth rates from a minimum of three successive growth curves had stabilised (S2 File, S2 Fig). A script written in the open source statistical software R [51] was used to process and analyse the growth rate data for each treatment [52] (S4 File). This objective approach improved the efficiency of data processing, given the large number of treatments (*n* = 174) and the duration of the culturing (~ 9 months) and removed potential bias or subjectivity when determining a growth rate from numerous data points.

### Growth rate versus light curve

The growth-light (μ-I) curves were modelled using the following function [53]:

$$\mu \;(d^{-1})=\mu_{max}\prime \;\cdot \;\left\{1-exp(\frac{-\alpha ∙(I-I_{c})}{{\mu_{max}}^{\prime }})exp(\frac{-\beta ∙I}{{\mu_{max}}^{\prime }})\right\}$$ (2)

where *μ*_*max*_*’* is the hypothetical maximum growth rate (d^-1^); *α* is the initial light-limited slope of the growth-light curve (d^-1^ (μmol photons m^-2^ s^-1^)^-1^); β is the parameter that characterises photoinhibition at supraoptimal light intensities (d^-1^ (μmol m^-2^ s^-1^)^-1^); I is the light intensity (μmol photons m^-2^ s^-1^); and I_c_ is the compensation light at which the light-limited growth rate extrapolates to zero (μmol photons m^-2^ s^-1^).

The achieved maximum growth rate (μ_max_), light at which growth is maximal (I_opt_), light-saturation parameter (I_k_), and light inhibition parameter (I_p_) were calculated from the fitted parameters as follows:

$$\mu_{max}=\mu_{max}\prime ∙\left(\frac{\alpha }{\alpha +\beta }\right)∙{\left(\frac{\beta }{\alpha +\beta }\right)}^{\frac{\beta }{\alpha }}$$ (3)

$$I_{opt}=\frac{\mu_{max}\prime }{\alpha }∙ln\;\left(\frac{\alpha +\beta }{\beta }\right)$$ (4)

$$I_{k}=\frac{\mu_{max}}{\alpha }$$ (5)

$$I_{p}=\frac{\mu_{max}}{\beta }$$ (6)

### Growth versus temperature curve

The growth-temperature (μ-T) curves were modelled using a newly formulated modified sine function, which returns estimates for T_min_, T_max_ and the maximum growth rate at T_opt_:

$$\mu \;(d^{-1})=\mu_{max}\;\cdot \;{\left\{\sin\left[\pi {\left(\frac{{T-T}_{min}}{T_{max}-T_{min}}\right)}^{\theta }\right]\right\}}^{\Phi }$$ (7)

where μ_max_ is the maximum growth rate (d^-1^); T is the temperature of the culture (°C); T_min_ is the minimum temperature limit for growth (°C); T_max_ is the maximum temperature limit for growth (°C); θ is a shape determining parameter, which alters the skewness; and Φ is a shape determining parameter, which alters the kurtosis.

The optimum temperature (T_opt_) is calculated from the fitted parameters as follows:

$$T_{opt}=\left[T_{min}+0.5^{\left(\frac{1}{\theta }\right)}∙(T_{max}-T_{min})\right]$$ (8)

The growth-light and growth-temperature curve fits for each CO_2_ or light treatment were fitted using a weighted non-linear squares algorithm, where weights were the reciprocals of the standard errors associated with the median growth rates. Standard errors were propagated when parameters (e.g. I_k_, T_opt_) were calculated from curve fit values (S3 Table). Further statistical analysis (Sigmaplot 11.0) was used to assess differences between CO_2_ and light treatments. When appropriate the data was log transformed to ensure normality, and a Two or Three-way ANOVA and *post hoc* Tukey test was applied on all of the growth rate data per light, CO_2_ treatment, as opposed to the single calculated curve fitted parameter values.

## Results

**Fig 1:**
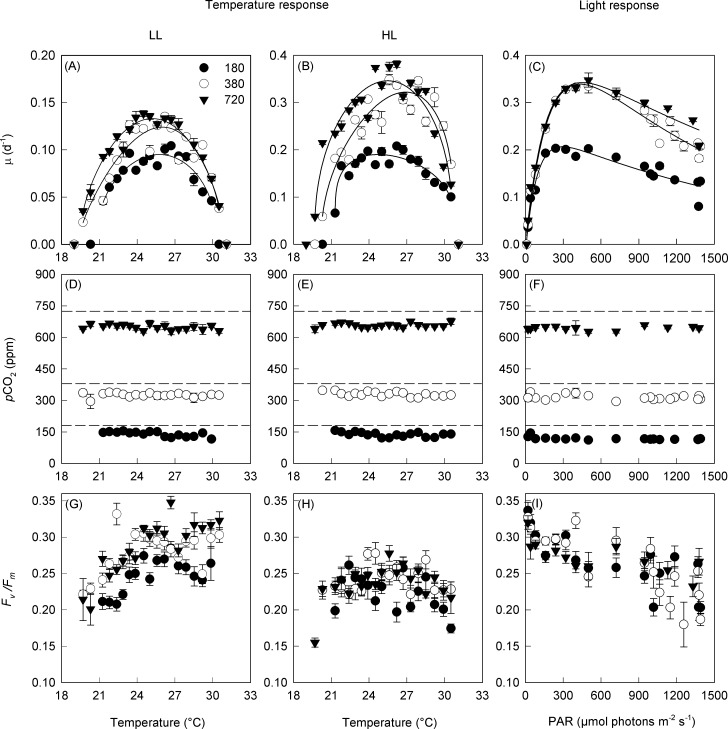
The median growth rate, and mean (± S.E.) *p*CO_2_ and maximum dark-adapted photochemical efficiency (*F*_*v*_*/F*_*m*_) of *Trichodesmium erythraeum* IMS101 when acclimated across a range of temperatures, light intensities, and at three target *p*CO_2_ concentrations (Low = 180 ppm, Mid = 380 ppm and High = 720 ppm) (174 treatments). For the temperature response: LL = 40 μmol photons m^-2^ s^-1^; HL = 400 μmol photons m^-2^ s^-1^. Note the dashed line represents the initial *p*CO_2_ concentration for each treatment culture once diluted (T = 0), while the data points are the final CO_2_ concentration post culturing.

Growth rates increased with increasing CO_2_ at all temperatures (Three-way ANOVA, Tukey post hoc test; P < 0.001). The growth responses were non-symmetrical around the optimum temperature for growth (T_opt_ ranging between 24.7 to 26.9°C); specifically the effect of *p*CO_2_ on growth was more pronounced at suboptimal than supraoptimal temperatures (Fig 1A and 1B). Growth rates at temperatures below T_opt_ were markedly lower at mid than high *p*CO_2_, whereas similar rates were observed at both mid and high *p*CO_2_ at temperatures above T_opt_.

The growth-temperature curves exhibited marked reductions of growth rate (μ) accompanying small changes in temperature near the lower and upper tolerance limits, and smaller rates of change of growth with changes of temperature closer to the optimum (Fig 1A and 1B). With the exception of the high light-low *p*CO_2_ treatment, all other curves exhibited negative skewness (θ > 1) (Table 3).

**Table 3:** The temperature dependent growth curve parameters (± S.E.) for *Trichodesmium erythraeum* IMS101 generated by fitting a five-parameter function to each of the two light (LL = 40 μmol photons m^-2^ s^-1^; HL = 400 μmol photons m^-2^ s^-1^) treatments at the three CO_2_ (Low = 180 ppm, Mid = 380 ppm and High = 720 ppm) treatments.

		Low CO_2_		Mid CO_2_		High CO_2_	
Parameters	Units	LL	HL	LL	HL	LL	HL
μ_max_	d^-1^	0.095 (±0.004)	0.190 (±0.006)	0.124 (±0.006)	0.322 (±0.012)	0.133 (±0.003)	0.346 (±0.012)
T_min_	° C	20.80 (±0.66)	21.29 (±0.01)	19.29 (±0.44)	20.18 (±0.13)	19.37 (±0.21)	19.66 (±0.05)
T_max_	° C	30.27 (±0.51)	30.85 (±0.50)	30.83 (±0.34)	30.76 (±0.37)	30.95 (±0.29)	30.66 (±0.19)
*w*	° C	9.47 (±0.83)	9.56 (±0.50)	11.54 (±0.56)	10.58 (±0.39)	11.58 (±0.36)	11.00 (±0.20)
Φ	−	0.38 (±0.21)	0.27 (±0.11)	0.54 (±0.19)	0.30 (±0.13)	0.55 (±0.12)	0.36 (±0.09)
θ	−	1.07 (±0.19)	0.68 (±0.11)	1.28 (±0.16)	1.53 (±0.35)	1.01 (±0.08)	1.09 (±0.12)
T_opt_	° C	25.75 (±0.69)	24.74 (±0.16)	26.00 (±0.46)	26.91 (±0.31)	25.20 (±0.23)	25.48 (±0.13)
*r*^2^		0.846	0.861	0.843	0.857	0.946	0.890

Units; μ_max_ (d^-1^), the maximum growth rate; T_min_ (° C), the minimum temperature limit for growth; T_max_ (° C), the maximum temperature limit for growth; *w* (° C), the thermal niche width for growth; Φ, the peakedness-shape determining factor (higher value = more peaked); θ, the skewness-shape determining factor (< 1 = positive skewness, > 1 = negative skewness). Both shape determining factors, Φ and θ, influence the shape of the curve without modifying μ_max_, T_min_ or T_max_.

The minimum temperature limit for growth (T_min_) was affected by both CO_2_ and light (Table 3). Under low light, T_min_ declined from 20.8 to 19.4°C between low and high CO_2_, whilst at high light the T_min_ declined from 21.3 to 19.7°C between low and high CO_2_. The maximum temperature limit for growth (T_max_) did not significantly vary between most treatments, averaging about 30.7°C (Table 3). The temperature niche width (*w* = Tmax—T_min_) was ~ 1.5 to 2°C smaller at low than at mid and high CO_2_.

There were significant differences in the growth rates between the low and high light treatment (40 versus 400 μmol photons m^-2^ s^-1^) at all CO_2_ treatments (Three-way ANOVA, Tukey post hoc test; P < 0.001). The maximum growth rate (μ_max_) at the optimum temperature increased by about 30% (low light) and 70% (high light) as CO_2_ increased from low to mid, and by an additional 7% (both low light and high light) from mid to high CO_2_ (Table 3).

A more comprehensive assessment of the light-dependence of growth rate was made at the optimum temperature of 26°C (Fig 1C). The initial slope (α) showed little response to *p*CO_2_, but the maximum growth rate (μ_max_) increased by 65% from about 0.21 d^-1^ at low CO_2_ to 0.33 d^-1^ at the mid and high CO_2_ (Table 4). The light intensity at which the highest maximum growth rate occurred (I_opt_) also increased with *p*CO_2_ from 290 μmol photons m^-2^ s^-1^ at low CO_2_ to 434 μmol photons m^-2^ s^-1^ at high CO_2_. The light-saturation parameter (I_k_) was much lower at low CO_2_ in comparison to both mid and high CO_2_ treatments (Table 4).

**Table 4:** The light-dependent growth curve parameters (± S.E.) for *Trichodesmium erythraeum* IMS101 generated by fitting a three-parameter P-I function to each of the three CO_2_ (Low = 180 ppm, Mid = 380 ppm and High = 720 ppm) treatments at optimal temperature (26°C).

Parameters	Units	Low CO_2_	Mid CO_2_	High CO_2_
μ_max_ʹ	d^-1^	0.252(±0.019)	0.507(±0.061)	0.437(±0.042)
μ_max_	d^-1^	0.207(±0.066)	0.330(±0.060)	0.330(±0.064)
α	d^-1^ (μmol photons m^-2^ s^-1^)^-1^	2.7·10^−3^(±4.9·10^−4^)	2.5·10^−3^(±2.4·10^−4^)	2.6·10^−3^(±3.2·10^−4^)
β	d^-1^ (μmol photons m^-2^ s^-1^)^-1^	1.3·10^−4^(±3.5·10^−4^)	3.6·10^−4^(±9.1·10^−5^)	2.1·10^−4^(±6.5·10^−5^)
I_k_	(μmol photons m^-2^ s^-1^)	78(±29)	131(±27)	125(±29)
I_p_	(μmol photons m^-2^ s^-1^)	1597(±4290)	921(±287)	1591(±585)
I_c_	(μmol photons m^-2^ s^-1^)	6.0(±5.5)	2.6(±4.9)	0.6(±5.8)
I_opt_	(μmol photons m^-2^ s^-1^)	290(±255)	419(±76)	434(±73)
*r*^2^		0.964	0.989	0.989

Units; μ_max_' (d^-1^), the hypothetical maximum growth rate in the absence of photoinhibition; μ_max_ (d^-1^), the achieved maximum growth rate; α (d^-1^ (μmol photons m^2^ s^-1^)), the initial slope of the growth-light curve; β (d^-1^ (μmol photons m^2^ s^-1^)), the photoinhibition slope of the growth-irradiance curve; I_k_ (μmol photons m^2^ s^-1^), the light-saturating parameter; I_p_ (μmol photons m^2^ s^-1^), the photoinhibition parameter.

The maximum photochemical efficiency of PSII (*F*_*v*_*/F*_*m*_) was greatest at T_opt_, and was significantly higher (Three-way ANOVA, Tukey post hoc test; P < 0.001) for low light than high light treatments (Fig 1G and 1H). In addition, *F*_*v*_*/F*_*m*_ increased as CO_2_ increased from low to mid and high CO_2_ (Three-way ANOVA, Tukey post hoc test; P < 0.001) (Fig 1G,H). At low light and for all CO_2_ treatments, *F*_*v*_*/F*_*m*_ decreased as temperature decreased from T_opt_ to T_min_, while there was no difference in *F*_*v*_*/F*_*m*_ (~ 0.3) as temperature increased above T_opt_ to T_max_ (Two-way ANOVA, Tukey post hoc test; P < 0.001). Conversely at high light, there was no significant difference of *F*_*v*_*/F*_*m*_ at the T_min_, T_opt_ or T_max_ values between CO_2_ treatments (Fig 1H). The maximum *F*_*v*_*/F*_*m*_ recorded (~ 0.35) was at T_opt_ and the lowest light intensity (20 μmol photons m^-2^ s^-1^) (Fig 1I).

## Discussion

Our key findings for *Trichodemisum* IMS101 are: (i) at T_opt_, CO_2_ affected μ_max_ but not the initial slope (α) of the growth-light curve; (ii) low CO_2_ constrained the thermal niche width more than low light; (iii) there was greater divergence in the temperature dependence of growth rate due to differences in CO_2_ below T_opt_ than above T_opt_; (iv) the maximum photosynthetic efficiency (*F*_*v*_*/F*_*m*_) was influenced more strongly by varying temperature and light than CO_2_; and (v) CO_2_ affected T_min_ while there was no effect of CO_2_ on T_max_. Here we discuss the physiological mechanisms that may explain these results.

### Light and CO_2_ dependencies of growth at T_opt_

The observed increase in μ_max_ with increasing *p*CO_2_ is qualitatively consistent with previous results [32,34], although there are slight quantitative differences in the percentage increase in growth between mid (~ 380 ppm) and high (~ 720 ppm) CO_2_ treatments.

Previous studies have attributed the low growth rates at low CO_2_ to high energy demands required to establish, maintain and operate a carbon concentrating mechanism (CCM), which in turn limits the energy available for N_2_ fixation, reducing growth [34,42,43]. Our observations of the effect of CO_2_ at light-saturation and optimal temperature support these findings, while the lack of variability in the light-limited initial slopes is inconsistent with this hypothesis because we would expect the initial slope to be lower at low CO_2_ if operation of the CCM imposed significant energetic cost. The lack of variation in the initial slopes were perhaps due to low data resolution (too few low-light data points) and the curve fitting process (more heavily weighted to the high-light data points). Consistent with this suggestion is our observation from the thermal response curves that growth rate at low light (40 μmol photons m^-2^ s^-1^) increased with increasing *p*CO_2_ by about 20% from low to mid CO_2_ with a further 10% increase from mid to high CO_2_.

Operation of the CCM is required to maintain high internal CO_2_ concentrations within the carboxysome to inhibit photorespiration. Conversely, at high CO_2_ concentrations, when the CCM is fully saturated, *Trichodesmium* spp. can down-regulate CCM activity and up-regulate other cellular processes (e.g. N_2_ fixation), which indirectly increases growth [40,42]. Although this is an attractive hypothesis, direct measurements of the magnitude and cost of operating the CCM in *Trichodesmium* spp. have not been reported. However, the effect on growth rate may be relatively small since the photon requirement for operating the CCM accounts for only 5–15% of the photon requirement for growth (S3 File, S2 Table).

The values of the light-saturation parameters (I_k_) were similar to previous observations [21], and exhibited a clear CO_2_ response, which was driven largely by the changes in μ_max_. The photoinhibition parameter (I_p_) was higher at high CO_2_ than mid CO_2_, indicating an increased capability at alleviating the effects of photoinhibition at high CO_2_, most likely attributed to CCM down-regulation and the up-regulation of photoprotective-related mechanisms. Interestingly, the I_p_ was also higher at low CO_2_ than mid CO_2_, which could be attributed to a low CO_2_ stress response triggering an up-regulation of photoprotective mechanisms. However, due to the standard error of the modelled slope of photoinhibition (β), the propagated error of the low CO_2_ I_p_ value is large, making a difference between the low and mid CO_2_ treatment a possible artefact of the curve fit.

### Minimum temperature for growth (T_min_)

The minimum temperature at which *Trichodesmium* spp. can grow is likely set by the ability to maintain anoxic conditions that are required to prevent nitrogenase inhibition [8]. Unlike heterocystous diazotrophs, *Trichodesmium* spp. do not possess a glycolipid barrier that prevents or reduces the diffusion of dissolved gases into the cell [8]. To prevent O_2_ inhibiting nitrogenase, *Trichodesmium* spp. uncouple photosynthesis from N_2_ fixation both temporally and spatially [54,55], maintains a high mitochondrial respiration rate [8,56] and may also use O_2_ scavenging processes (Mehler reaction and hydrogenase activity) to maintain an anoxic state within the diazocytes [34,57,58]. Stal [8] calculated that respiration would not be able to maintain anoxia at T < 17°C, which is slightly below the T_min_ of 19 to 21°C that we (Table 3) and others [26] have observed. The higher T_min_ that we observed at low CO_2_ may reflect lower availability of substrate for respiration due to lower photosynthesis rate, or it may reflect the higher metabolic demand for operation of a carbon concentrating mechanism, which diverts energy that would otherwise be available to fuel N_2_ fixation.

### Supra-optimal temperatures for growth (T_opt_ to T_max_)

The shape of the growth curve from T_opt_ to T_max_ exhibited two distinct sections, where growth steadily decreases to ~ 29°C, before plummeting to zero at T_max_. This two-part decline in growth was most pronounced at low light-low CO_2_, which may in part be due to an enhanced CCM activity limiting the efficacy of certain heat stress processes.

The maximum temperature limit for growth (T_max_) was similar across all light and CO_2_ treatments. The primary targets of thermal damage in vascular plants include the oxygen evolving complex along with the associated cofactors in photosystem II (PSII), the activity of the carboxylating enzyme Rubisco and the ATP generating system [59]. The maintenance of high values of *F*_*v*_*/F*_*m*_ at temperatures above T_opt_ under light-limiting conditions indicates that PSII was not damaged by high temperature in these benign low light conditions irrespective of *p*CO_2_. In contrast, under light-saturated conditions, *F*_*v*_*/F*_*m*_ peaked near T_opt_ under all *p*CO_2_ treatments.

Reduced growth rates caused by temperatures exceeding T_opt_ may perhaps be due to the inhibition of Rubisco activity or an increase in the ratio of oxygenation to carboxylation by Rubisco. In comparison to other photosynthetic enzymes, Rubisco has a low turnover rate requiring high concentrations to maintain a sufficient rate of photosynthesis. Rubisco activase has a lower maximum temperature tolerance than Rubisco itself. Therefore, if the temperature exceeds the temperature limit to which an organism is adapted, the activity of Ruisco activase is severely reduced and unable to offset the rate of Rubisco deactivation, reducing or inhibiting photosynthesis [60–62]. Previous studies have shown Rubisco activase activity to significantly decrease at ~ 30°C, with maximum inefficiency occurring at 45°C [61]. Although the T_max_ values reported here were ~ 31°C, it is likely that the concentration, substrate affinities and the temperature adaptations of *Trichodesmium’s* Rubisco activase and Rubisco itself would differ from terrestrial plants and other cyanobacteria.

Rubisco in *Trichodesmium* spp. is characterised by a low affinity for CO_2_ relative to ambient CO_2_ concentrations [63–65]. As temperatures increase, the solubility of CO_2_ decreases more rapidly than that of O_2_. As such, increasing temperature favours the oxygenation of Rubisco (photorespiration), which reduces the rate of 3-Phosphoglycerate production and requires significant amounts of energy and reductant to process the NH_3_ and potentially enzyme-inhibiting (i.e. 2-P glycolate) by-products. The solubility of CO_2_ and O_2_ within the carboxysomes is principally governed by temperature and not by light. Despite this there was a low light, low CO_2_ integrated effect on T_max_. A possible explanation for this observation could be that at low CO_2_, the CCM is up-regulated and functioning at maximum efficiency to maintain a sufficient internal CO_2_ concentration within the carboxysomes. At low light, photosynthetic rates are reduced, limiting the amount of energy that can be invested into CCM-related proteins. Thus, the combination of low light with low CO_2_ may make cells more susceptible to photorespiration, particularly at elevated temperatures where the solubility of CO_2_ is reduced.

To recap, we suggest that the observed two-part decline in growth between the T_opt_ and T_max_ values are due to two processes, photorespiration and Rubisco inhibition. The former primarily mediated by the temperature-driven changes to the solubility of O_2_ and CO_2_, where the response is compounded by other co-limiting factors (i.e. low CO_2_ and light); and the latter solely mediated by the temperature-driven response on enzyme kinetics, and is determined by *Trichodesmium’s* thermal tolerance and is not influence by other abiotic factors.

### Potential effects of future climate change on the biogeography of *Trichodesmium*

Although temperature, CO_2_ and light intensity place limits on the potential for *Trichodesmium* growth, whether this potential is achieved depends on the availability of limiting nutrients (e.g. P, Fe). *Trichodesmium* spp. are most abundant in regions of the sea that receive high inputs of Fe via deposition of dust transported from arid source regions [66]. This may reflect *Trichodesmium*’s high metalloenzyme inventory, which suggests that iron, instead of phosphorus, may be the key nutrient in constraining *Trichodesmium* growth and productivity in the present and future oceans [67]. Increases in the supply of Fe to the ocean via increased dust deposition as desertification increases in a warmer climate may also favour *Trichodesmium* spp. [68–70]. However, this advantage may be negated if increases in the dust load occur where elevation of temperature above 26°C (T_opt_) inhibits growth of *Trichodesmium*.

Climate models indicate that the water column in oligotrophic regions of the ocean will become more stratified as global sea surface temperatures rise due to global warming, decreasing vertical mixing and thereby limiting the supply of new nutrients from the deep ocean [5]. The documented increase of water column transparency (decline of phytoplankton chlorophyll) in low to mid latitude oligotrophic oceans over the past 120 years suggests that phytoplankton abundance has declined. This is presumed to be due to an increase in vertical stratification [71], although this century scale observation does not necessarily appear to explain local changes of chlorophyll and stratification on shorter time scales [72]. Increased water column stratification with lower inorganic N concentrations in the surface mixed layer should give photosynthetic diazotrophs such as *Trichodesmium* spp. a competitive advantage over other phytoplankton [73]. However, exposure to high light intensities in shallower near surface mixed layers may reduce growth by requiring more energy be used for prevention or repair of damage due to photoxidative stress [74]. Although vertical inorganic P fluxes will also decline as the water columns become more stable, *Trichodesmium* spp. have several adaptations that allow it to exploit P-limited environments. These include an extracellular hydrolysis of dissolved organic P (DOP) by alkaline phosphatase activity [75], which can provide an additional source of P for growth. *Trichodesmium* spp. can also upregulate synthesis of proteins associated with high-affinity transport and hydrolysis of phosphonate compounds [76]. To date, this pathway is absent from other sequenced marine cyanobacterial genomes, and thus represents a unique adaption for scavenging and hydrolysing phosphorus compounds from organic sources, and growing in otherwise P-limited regions. In addition, *Trichodesmium* spp. may be capable of mining phosphate by increasing their density by carbohydrate production facilitating sinking to below the nutricline, where they take up phosphate before using gas vesicles to increase buoyancy enabling a return to the surface of the euphotic zone [77,78]. Finally, the additional N and energy investments required for exoenzymatic breakdown of DOP appears to give N_2_ fixers a competitive advantage in oligotrophic regions [79].

**Fig 2:**
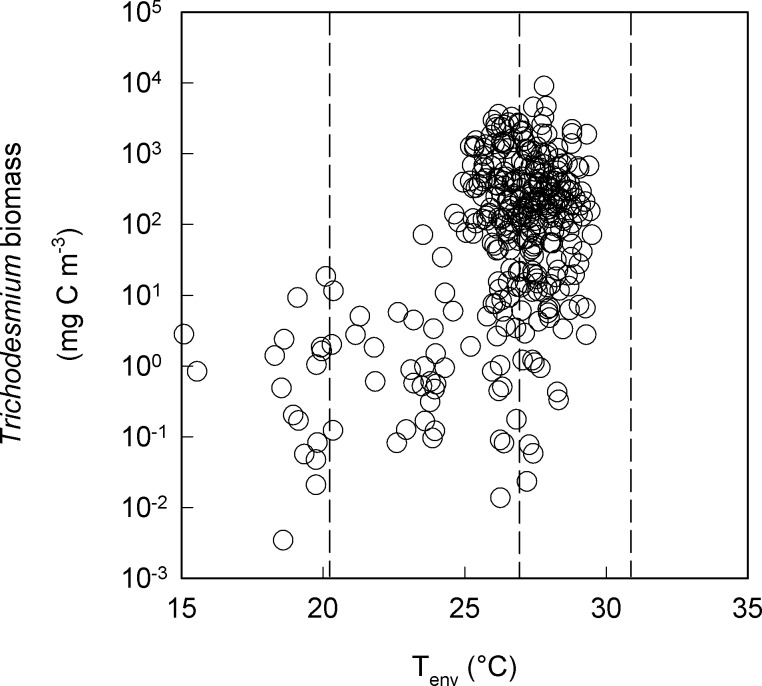
*Trichodesmium* is found most frequently and its biomass is highest where water temperature is supra-optimal for growth of IMS101. The dashed lines represent the minimum (T_min_), optimal (T_opt_) and maximum (T_max_) temperature limits for *Trichodesmium* IMS101 growth for the mid CO_2_ (~ 380 ppm), high-light treatment. Data from the MARine Ecosystem Data archive (https://doi.pangaea.de/10.1594/PANGAEA.774851).

The thermal niche of *Trichodesmium*, which is confined to a relatively narrow temperature range from 19–30°C, is a key factor that sets the limits of its geographic distribution. It is clear that temperature plays a pivotal role in constraining *Trichodesmium’s* global distribution as i), peak abundance for *Trichodesmium* sp. occurs in regions that are supra-optimal temperature for growth of IMS101 (Fig 2) and other *Trichodesmium* isolates (e.g. GBRTRL 1101, KO4-20, RLI or 2175) that have been investigated [27,28] and ii), the growth response to temperature is negatively skewed. Growth rates decrease significantly with a 3 to 4°C increase above the optimal (26°C). The areal distribution of *Trichodesmium* spp. is predicted to increase as the 20°C isotherm slowly shifts pole-wards [80,81]. None-the-less, the negative effects of increased temperature and light at low latitudinal regions where temperature already exceeds T_opt_ may lead to a contraction of the range at low latitudes. How future increases of SST will influence the distribution will depend on the capacity for *Trichodesmium* spp. to adapt by increasing its upper thermal tolerance limit.

Increases in atmospheric CO_2_ are transmitted to the ocean, increasing the dissolved CO_2_, and causing ocean pH and carbonate concentration to decline and bicarbonate concentration to increase. Recent research suggests that ocean acidification has greater potential to increase phytoplankton growth rates in areas of the ocean where temperature is equal to or less than the T_opt_ [82]. In regards to *Trichodesmium* IMS101, previous research indicates that increasing CO_2_ above 380–400 ppm can lead to modest increases in *Trichodesmium* growth rate [33,34,38–40,42,43], some studies have reported declines in growth at elevated CO_2_ [36,37] and others no change [32,35]. Our study suggests that ocean acidification will have a small to negligible effect for growth at the supraoptimal temperatures above 27°C. Differing growth responses to elevated CO_2_ between key phytoplankton types could cause sufficient shifts in competitive fitness to alter community structure [83]. Thus, the effect of ocean acidification, albeit indirectly, may still play a role in constraining *Trichodesmium’s* global distribution.

## Supporting Information

S1 Fig *Trichodesmium’s* three growth phases (i.e. lag, exponential and stationary) defined by changes in the dark-adapted minimum fluorescence (*F*_*o*_) over time (solid line). All experimental cultures were semi-continuously cultured at balanced growth at the lower section of the exponential growth phase (dashed line). The highest *F*_*o*_ value a culture achieved prior to dilution was ~ 20% of the stationary phase *F*_*o*_ value.
(TIF)

S2 Fig The three criteria applied to each treatment to generate a median growth rate (Treatment reported here: high CO_2_ (~ 720 ppm), 400 μmol photons m^-2^ s^-1^). The R code was applied to the full data set of each treatment (A). Criterion 1 identifies and discards data associated with crashed cultures (large circles) or lagged growth (B). Criterion 2 then identifies and discards individual data points (small circles) which, if incorporated, would significantly alter the gradient of a slope (C). Finally, criterion 3 identifies and discards slopes (dashed lines) associated with acclimation (D). The remaining slopes are associated with balanced growth, and were used to calculate a median growth rate (E).
(TIF)

S1 File Control of inorganic carbon chemistry in the culture medium. (DOCX)

S2 File Criteria employed to determine fully acclimated growth rate. (DOCX)

S3 File Cost of CCM on *Trichodesmium* growth. (DOCX)

S4 File R code to implement determination of fully acclimated growth rate. (DOCX)

S1 Table The range of the experimental treatments used to measure *Trichodesmium*’s light and temperature growth curves. Temperature response growth conditions; low (~ 180 ppm), mid (~ 380 ppm) and high (~ 720 ppm) CO_2_, 40 μmol photons m^-2^ s^-1^ (LL) and 400 μmol photons m^-2^ s^-1^ (HL), ranging between 18–31°C. Light response growth conditions; low (~ 180 ppm), mid (~ 380 ppm) and high (~ 720 ppm) CO_2_, 26°C, ranging between 20–1400 μmol photons m^-2^ s^-1^). A circle (O) represents a growing culture; a cross (X) represents a condition where growth did not occur; a dash (-) represents a condition that was not used for culturing.
(DOCX)

S2 Table Calculation of the minimum energetic requirement for growth of *Trichodesmium*. Footnotes to S2 Table. ^a^ CO_2_ fixation to carbohydrate in the Calvin cycle according to the following stoichiometry. CO_2_ + 3 ATP + 2 NADPH → CH_2_O + H_2_O + 3ADP +3 P_i._ The photon requirement (9 photons/CO_2_ fixed) is from Raven et al. [84]. ^b^ Carbon concentrating mechanism where the only energised step is the influx of HCO_3_^-^ at one membrane between the medium and Rubisco. Lower value assumes no leakage, whereas the upper value assumes leakage rate equals to the rate of photosynthesis [84]. ^c^ The energetic cost of N_2_ fixation was calculated assuming complete recycling of H_2_ to recover ATP was calculated from the following stoichiometry: N_2_ + 6 H^+^ + 6 e^-^ + 13 ATP → 2 NH_3_ + 13 ADP + 13 P_i._^d^ The cost of ammonium assimilation into amino acids is 1 ATP/NH_3_ and 1 NADPH (2 reducing equivalents) assimilated via GOGAT. Protein synthesis would require an additional 4 ATP per peptide bond formed. ^e^ Based on a typical photosynthetic quotient of 1.2 O_2_ evolved per CO_2_ fixed for algae growing with ammonium as the inorganic N source. This accounts for the more reduced state of lipids and proteins relative to carbohydrates. ^f^ Total cost of synthesising 1 unit of C-biomass assumes a Redfield C:N ratio of 106C:16N and that protein accounts for all of the cell N. ^g^ Photon requirements were calculated based on 1/3 ATP generated per photon absorbed during linear photosynthetic electron transfer from H_2_O to O_2_, with the additional ATP requirement from provided either by LPET from H_2_O to H_2_O (water-water cycle) with 1/3 ATP generated per photon absorbed (higher estimate) or by cyclic photosynthetic electron transfer around photosystem I with 1 ATP generated per photon absorbed.
(DOCX)

S3 Table The summary of rules for error propagation. Key; *x* is the calculated value, *σ*_*x*_ is the calculated error of uncertainty; *a*, *b* and *c* are known quantities; *σ*_*a*_, *σ*_*b*_ and *σ*_*c*_ are errors of uncertainty for *a*, *b* and *c*, respectively; y is a constant with no measure of uncertainty.
(DOCX)
