# Supplementary material for: A Key Marine Diazotroph in a Changing Ocean: The Interacting Effects of Temperature, CO2 and Light on the Growth of Trichodesmium erythraeum IMS101
Source: PLoS One. 2017 Jan 12;12(1):e0168796. doi: 10.1371/journal.pone.0168796 (PMC5230749; doi:10.1371/journal.pone.0168796)
Supplement: S1 File — (DOCX) [file pone.0168796.s003.docx]

**S1 File. Control of inorganic carbon chemistry in the culture medium.**

A single batch (25 L) of filter-sterilised (0.25 µm pore) YBCII media [[1](#_ENREF_1)] was made and stored in acid-washed, autoclaved Duran bottles (no headspace). The inorganic carbon chemistry of each bottle was quantified immediately before use from measurements of TCO_2_ and pH taken at 26 °C, using *CO2SYS* [[2](#_ENREF_2)] to calculate inorganic carbon speciation, *p*CO_2_ and alkalinity. TCO_2_ was measured using a Shimadzu TOC-V Analyser & ASI-V Autosampler) and pH was measured with a Thermo Scientific Orion Ross Ultra pH Electrode (EW-05718-75) calibrated with newly made (< 2 weeks) artificial seawater buffers (TRIS and AMP) prior to use [[3](#_ENREF_3)].

The carbonate chemistry of the medium was manipulated by bubbling with a CO_2_-air mixture as opposed to using strong acids or bases. This method better resembles future conditions where the *p*CO_2_ and TCO_2_ will change whilst maintaining a constant alkalinity concentration [[4](#_ENREF_4)]. Inorganic carbon speciation is temperature-dependent but the total alkalinity and TCO_2_ concentration is independent of temperature. Therefore, the inorganic carbon chemistry of the medium was adjusted for each culture tube prior to sub-culturing. The following information was entered into the *CO2SYS* macro:

Input conditions; the salinity of the YBCII medium (35 psu), the P concentration in the YBCII medium (42.515 µM P (kg ASW)^-1^) and the growth temperature of the tube being diluted (e.g. 20 °C):

Output conditions; the temperature at which the pH was being measured (i.e. 26 °C):

Data; the previously quantified alkalinity concentration (µM kg ASW for *CO2SYS*) and the target *p*CO_2_ concentration (i.e. 180, 380 or 720 µatm).

The computation provided the exact pH (at 26 °C) required to achieve the target *p*CO_2_ at a specific growth temperature. This pH was then used as a target value on a pH stat system, which allowed the pH to be adjusted by either bubbling with short pulses of CO_2_ enriched air (10 % CO_2_ cylinder) or CO_2_ free air. In addition to pH, the computation also produced initial (pre-culturing) values for TCO_2_, HCO_3_, CO_3_ and CO_2_ at the specific growth temperature. This initial value of total alkalinity was assumed to remain constant throughout culturing, and is used latter in the calculation of the carbonate chemistry in the culture tubes at the point when cells were transferred.

A post-culturing measurement of pH was taken at 26 °C. Using the same constants, the following information was entered into *CO2SYS*:

Input conditions; the salinity of the YBCII medium (i.e. 35), the P concentration in the YBCII medium (42.515 µM kg ASW for *CO2SYS*) and the growth temperature of the treatment (e.g. 20 °C):

Output conditions; the temperature at which the pH was being measured (i.e. 26 °C):

Data; the initial alkalinity concentration which is temperature independent and the post-culturing pH measurement.

The computation calculated the inorganic carbon chemistry of the YBCII medium at the growth temperature of the treatment (e.g. 20 °C). This included post-culturing values of TCO_2_, HCO_3_, CO_3_, CO_2_ and *p*CO_2_. Note, all *p*CO_2_ data calculated in *CO2SYS* was in units of µatm but is reported here as ppm.

**References.**

1. Chen YB, Zehr JP, Mellon M (1996) Growth and nitrogen fixation of the diazotrophic filamentous nonheterocystous cyanobacterium Trichodesmium Sp. IMS 101 in defined media: evidence for a circadian rhythm. Journal of Phycology 32: 916-923.

2. Lewis E, Wallace D (1998) CO2SYS Program. Carbon Dioxide Information Analysis Center, Oak Ridge National Laboratory Environmental Sciences Division, Oak Ridge, Tennessee.

3. Dickson AG (1993) pH buffers for sea water media based on the total hydrogen ion concentration scale. Deep Sea Research Part I: Oceanographic Research Papers 40: 107-118.

4. Gattuso J-P, Lavigne H (2009) Perturbation experiments to investigate the impact of ocean acidification: approaches and software tools. Biogeosciences Discussions 6.
