# Supplementary material for: A Key Marine Diazotroph in a Changing Ocean: The Interacting Effects of Temperature, CO2 and Light on the Growth of Trichodesmium erythraeum IMS101
Source: PLoS One. 2017 Jan 12;12(1):e0168796. doi: 10.1371/journal.pone.0168796 (PMC5230749; doi:10.1371/journal.pone.0168796)
Supplement: S2 File — (DOCX) [file pone.0168796.s004.docx]

**S2 File. Criteria employed to determine fully acclimated growth rate.**

*In vivo* fluorescence was used as an index of biomass for determining growth rate [[1](#_ENREF_1)]. The growth rate of each test tube culture was quantified from daily (between 09:00 to 10:30) measurements of fluorescence (*F_o_*) on dark-adapted cells (20 minutes) using a FRRfII Fastact Fluorometer (Chelsea Technologies Group Ltd, UK). The FRRfII parameters were optimised prior to the experiment to ensure that a saturating fluorescence curve was achieved for both low (post-dilution) and high (pre-dilution) cell density cultures.

Cultures were kept at the lower section of the exponential growth phase by periodic dilution with YBCII medium (S1 Fig) to avoid nutrient limitation, self-shading and minimize CO_2_ drift [[2](#_ENREF_2)]. All tubes were gently inverted twice a day to minimise the possibility of the trichomes aggregating at the meniscus. Subject to the temperature and CO_2_, high light cultures were usually diluted every fourth to fifth day, while low light cultures every tenth to twelfth day. When *F_o_* declined at an extreme growth condition (e.g. high temperature), three attempts were made to re-grow at that treatment, using culture from the closest growth condition.

Growth rates were calculated by fitting a linear regression to the ln(*F_o_*) values for each growth curve. A script (S4 File) written in the open source statistical software R [[3](#_ENREF_3)] was used to process and analyse the growth rate data for each treatment. This objective approach improved the efficiency of data processing and removed potential bias or subjectivity when determining a growth rate from numerous data points. The raw data (date, time, *F_o_* value etc) was converted to csv format and imported into R.

Three criteria were used to ensure that fully acclimated growth rate were returned from the statistical analysis. The first criterion selects growth rate slopes which terminated with a minimum of 3 increasing *F_o_* values, which removed data associated with crashed cultures. The second criterion analysed the data points within the remaining growth curves. Data points were used where the largest sequence of consecutive observations, which had to include the final observation of the *F_o_* gave a multiple correlation coefficient, R^2^, for a linear fit through the logged growth rate greater than R^2^_crit_ (R^2^_crit_ = 99%). This insured that slopes were calculated from data that were collinear, thus removing any data point at the start of a useful slope which, if incorporated, would significantly decrease the calculated growth rate. The third criterion compared each growth rate slope against the common slope (median of all slopes). If a slope was significantly different (P < 0.001) from the common slope, then it was discarded. If multiple slopes were significantly different from the common slope, then the slope with the most difference was discarded. All remaining slopes were re-tested and criterion three repeated until there were no discordant slopes, or two slopes remained. If the two slopes were significantly different, then no median growth rate would have been produced for that treatment; however, this was never the case. This process yielded slopes associated with balanced growth after full acclimation to the prevailing abiotic condition (S2 Fig).

**References.**

1. Wood AM, Everroad R, Wingard L (2005) Measuring growth rates in microalgal cultures. Algal Culturing Techniques: 269-285.

2. Barcelos e Ramos J, Biswas H, Schulz KG, LaRoche J, Riebesell U (2007) Effect of rising atmospheric carbon dioxide on the marine nitrogen fixer *Trichodesmium*. Global Biogeochemical Cycles 21: GB2028.

3. R*-*Development*-*Core*-*Team (2014) R: A language and environment for statistical computing. Vienna, Austria.
