# Supplementary material for: A Key Marine Diazotroph in a Changing Ocean: The Interacting Effects of Temperature, CO2 and Light on the Growth of Trichodesmium erythraeum IMS101
Source: PLoS One. 2017 Jan 12;12(1):e0168796. doi: 10.1371/journal.pone.0168796 (PMC5230749; doi:10.1371/journal.pone.0168796)
Supplement: S3 File — (DOCX) [file pone.0168796.s005.docx]

**S3 File. Cost of CCM on *Trichodesmium* growth.**

Raven et al. [[1](#_ENREF_1)] calculated the minimum photon cost for operating cyanobacterial CCMs where CO_2_ enters the cells by passive diffusion across the plasma membrane through selective protein channels. The CO_2_ is then converted into HCO_3_^-^ by a specific NAD(P)H–PQ oxidoreductase located in the thylakoid membrane thus allowing intracellular concentrations of HCO_3_^-^ to increase above those in the surrounding bulk medium. The HCO_3_^-^ then enters the carboxysome, where it is converted back to CO_2_ by carbonic anhydrase prior to fixation by Rubisco. Depending on the assumptions made regarding the photon efficiency of the PSI driven NAD(P)H–PQ oxidoreductase and the magnitude of the leakage of CO_2_ away from Rubisco, the calculated photon requirement ranged from 0.5 to 2 photons per CO_2_. This represents 5-20% of the cost of CO_2_ fixation. Perhaps more informative is a comparison of the photon cost of the CCM with the photon cost of N_2_ fixation. Based on a Redfield C:N of 106:16, and the ATP and reductant requirements for nitrogen fixation, the minimum photon requirement of 1.7-3 photons per CO_2_ fixed into biomass is obtained (S2 Table). This suggests that there may be some room for an energetic trade-off between the costs of CCM and N_2_ fixation. However, the effect on growth rate is likely to be small since the photon requirement for operating the CCM accounts for only 5-15% of the photon requirement for growth (S2 Table).

**References.**

1. Raven JA, Beardall J, Giordano M (2014) Energy costs of carbon dioxide concentrating mechanisms in aquatic organisms. Photosynthesis Research 121: 111-124.
