# Supplementary material for: A Key Marine Diazotroph in a Changing Ocean: The Interacting Effects of Temperature, CO2 and Light on the Growth of Trichodesmium erythraeum IMS101
Source: PLoS One. 2017 Jan 12;12(1):e0168796. doi: 10.1371/journal.pone.0168796 (PMC5230749; doi:10.1371/journal.pone.0168796)
Supplement: S4 File — (DOCX) [file pone.0168796.s006.docx]

**S4 File. R code to implement determination of fully acclimated growth rate.**

plot.new() #New plot page

par(mfrow=c(3,2)) #6 rows and 2 columns of plots on one page

par(mar=c(4,4.5,2,2)) #Sets the margins around the plots

RSQCRIT=0.99;pCRIT=0.01

title=c("180LL","180HL","380LL","380HL","720LL","720HL")

temps=c(19.0, 19.7,20.3, 21.3, 21.8, 22.4, 22.9, 23.4, 23.9, 24.5, 25.0, 25.6, 26.2, 26.7, 27.3, 27.9, 28.5, 29.2,

29.9, 30.5, 31.1)

ntemps=length(temps)

ngroup=6

finisht=0

allsmax=c(1:ngroup);alltmin=c(1:ngroup);alltmax=c(1:ngroup);weightedrss=c(1:ngroup);allalpha=c(1:ngroup);allbeta=c(1:ngroup)

nrecords=120 #The total number of temperature/CO2 combinations including zeroes at end of each run

allslopeplus=c(1:nrecords)

allseplus=c(1:nrecords)

alltemp=c(1:nrecords)

ALLWEIGHT=c(1:nrecords)

for (trial in 1:ngroup){

if(trial==1){id=c('E1','E2','E3','E4','E5','E6','E7','E8','E9','E10','E11','E12','E13','E14','E15','E16');temp1=4;temp2=19;SKIP=1}

if(trial==2){id=c('F1','F2','F3','F4','F5','F6','F7','F8','F9','F10','F11','F12','F13','F14','F15','F16','F17');temp1=4;temp2=20;SKIP=1}

if(trial==3){id=c('A-1','A0','A1','A2','A3','A4','A5','A6','A7','A8','A9','A10','A11','A12','A13','A14','A15','A16','A17');temp1=2;temp2=20;SKIP=1}

if(trial==4){id=c('C0','C1','C2','C3','C4','C5','C6','C7','C8','C9','C10','C11','C12','C13','C14','C15','C16','C17');temp1=3;temp2=20;SKIP=2}

if(trial==5){id=c('B-1','B0','B1','B2','B3','B4','B5','B6','B7','B8','B9','B10','B11','B12','B13','B14','B15','B16','B17');temp1=2;temp2=20;SKIP=1}

if(trial==6){id=c('D0','D1','D2','D3','D4','D5','D6','D7','D8','D9','D10','D11','D12','D13','D14','D15','D16','D17','D18');temp1=2;temp2=20;SKIP=1}

nid=length(id)

slope=matrix(0,nid,1);se=matrix(0,nid,1)

########## START TEMPERATURE LOOP ##########

for (i in 1:nid){ #Looks at the temperatures one at a time

day=5;lnf=9;growth=13

dataset=paste('/Users/gupton/Dropbox/Trichodesmium/',title[trial],'/',id[i],'.csv',sep="")

data=read.csv(dataset,skip=SKIP)

T=paste(data[,1],data[,2]) #Combines the timing columns

dates=strptime(T,"%d/%m/%Y %H:%M:%S") #Forms a single decimal time variable (in seconds)

set=array(NA,dim(data)[1],1)

wstart=which(data[,growth]=="-") #Scans down column to find the start points

nstarts=length(wstart) #Number of segments

dstart=data[wstart,day] #Find the corresponding start days

nobs=dim(data)[1]

nused=c(1:nobs)*0;rsqlast=c(1:nobs)*0;wfinish=wstart*0 #Initialisation

wfinish[1:(nstarts-1)]=wstart[2:nstarts]-1 #Locates the ends of the segments

wfinish[nstarts]=nobs

finishdays=data[wfinish,day] #Finds the corresponding finish days

WEIGHT=matrix(1,nobs,1) #Initially all observations are given weight 1

for (j in 1:nstarts){

set[wstart[j]:wfinish[j]]=j #Assigns an index that identifies the segment within which an observation lies

}

set=factor(set) #Makes the segments have names rather than values

########## SCREENING SECTIONS ########## BAD=matrix(1,nstarts,1);RSQ=matrix(0,nstarts,1);SLOPE=matrix(1,nstarts,1) #Initialising

for(k in 1:nstarts){ #Looks at the segments one at a time

BIT=c(wstart[k]:wfinish[k]) #Identifies the data in a segment

y=data[BIT,lnf];x=data[BIT,day];length=wfinish[k]-wstart[k]+1

if(length==1){ #A segment of length 1 gives no info re slope, so give weight 0

BAD[k]=-2

WEIGHT[BIT]=0

}

if(length>1){

for(kk in length:2){ #Step back through a segment, looking for an decrease

if(y[kk]<y[kk-1]){

for(l in wstart[k]:(wstart[k]+kk-1)){

WEIGHT[l]=0 #If a decrease then give a zero weight to all previous observations

}

}

}

sumweight=sum(WEIGHT[BIT]) #Check at least 3 retained observations

if(sumweight<3){

BAD[k]=-1 #If not, give a segment weight zero

WEIGHT[BIT]=0

}

}

if(BAD[k]==1){ # For segments passing scrutiny

first=wstart[k];RSQ[k]=0

while(RSQ[k]<RSQCRIT&first<wfinish[k]){

BIT=c(first:wfinish[k])

y=data[BIT,lnf];x=data[BIT,day]

model=lm(y~x,weight=WEIGHT[BIT]) #Fit a linear function to the correct part of segment

RSQ[k]=summary(model)$r.squared

if(model$coeff[2]==0){RSQ[k]=1.00}

SLOPE[k]=model$coeff[2]

if(RSQ[k]<RSQCRIT|length(BIT)<3){ #If Rsq not large enough or fewer than 3 retained observations, give a segment zero weight

WEIGHT[first]=0

first=first+1

}

}

}

}

# ###########################################################################

y=data[,lnf];x=as.numeric(dates)/(60*60*24)

lotused=which(BAD==1);nlotused=length(lotused)

if(nlotused>1){

allok=0 #allok=1 if final model found

while(allok==0&sum(BAD)>2){

prob=matrix(1,nstarts,1)

for(k in 1:nstarts){

MOST=matrix(1,nobs,1)

BIT=c(wstart[k]:wfinish[k])

MOST[BIT]=2 #All data given index 1 except for bit under scrutiny (index 2)

modelBIT=lm(y~x*MOST+set,weight=WEIGHT) #Model allows for two slopes (via MOST) and multiple intercepts (via set)

row=dim(summary(modelBIT)$coeff)[1]

prob[k]=summary(modelBIT)$coeff[row,4] #Tail probability associated with significance of slopes not varying

}

allok=1 #Will be final model if all tail probabilities are OK

w=which(BAD==1);m=min(prob[w])

if(m<pCRIT){ #Smallest tail probability is too small

allok=0

wm=which(prob==m&BAD==1) #Determine which segment(s) have this small probability

BAD[wm]=0

len=length(wm)

for (jj in 1:len){

BIT=c(wstart[wm[jj]]:wfinish[wm[jj]])

WEIGHT[BIT]=0 #Set weight to zero for this segment (and therefore repeat the `while' loop

}

}

}

}

if(nlotused>0){

model=lm(y~x+set,weight=WEIGHT) #Final model, with only one slope

RSQALL=round(100*summary(model)$r.squared,1)

slope[i]=model$coeff[2] #Slope found

se[i]=summary(model)$coeff[2,2] #Associated se

nobsused=sum(WEIGHT) #Number of observations used in slope determination

lotused=which(BAD==1) #Which segments used in final fit

observations=c(1:nobs)*WEIGHT

used=which(observations>0)

file= '/Users/gupton/Dropbox/Trichodesmium/TRICHoutput'

write(c('Trial:',title[trial],'Temp:',format(temps[temp1+i-1],nsmall=1),'Rsq=',format(RSQALL,nsmall=1),'Rate=',format(round(slope[i],4),nsmall=4),'se=',format(round(se[i],4),nsmall=4),'Using the following',format(nobsused,nlarge=2),'observations:',used),file,ncolumns=50,append=TRUE)}

if(nlotused==0){

print(paste('No useful information at this temperature (',format(temps[temp1+i-1],nsmall=1),')'))

slope[i]=NA}

}

plot(temps[temp1:(temp1+nid-1)],slope,xlab="Temperature (degrees C)", ylab="Growth rate (per day)",xlim=c(temps[1],temps[ntemps]),ylim=c(0,0.45),main=title[trial])

for(j in 1:nid){lines(c(temps[j+temp1-1],temps[j+temp1-1]),c(slope[j]+se[j],slope[j]-se[j]),col="red")}

points(temps[temp1-1],0);points(temps[temp1+nid],0)

########## FITTING SECTION ##########

temp=temps[(temp1-1):(temp2+1)]

f=function(t,smax,tmin,tmax,alpha,beta){s=t;diff=max(tmax-tmin,0.0001);for(i in 1:length(t)){num=max((t[i]-tmin),0.0);

z= pi*(num/diff)^alpha;s[i]=max(sin(z),0.0)};smax*(s^beta)}

len=length(temp)

slopeplus=c(1:len);seplus=slopeplus

for (jj in 2:(len-1)){slopeplus[jj]=slope[jj-1];seplus[jj]=se[jj-1]

slopeplus[1]=0;slopeplus[len]=0;seplus[1]=0.000001;seplus[len]=0.000001

WEIGHT=as.vector(1/seplus)

model=nls(as.vector(slopeplus)~f(as.vector(temp),smax,tmin,tmax,alpha,beta), weights=WEIGHT, algorithm='port',

start=list(smax=max(slope)/2,tmin=temp[1]+0.1,tmax=temp[len]-0.1,alpha=1.2,beta=0.6),lower=c(0.5*max(slope),temp[1],temp[len-1],0.1,0.1),upper=c(max(slope),temp[2]-0.01,temp[len],2,2))

print( summary(model) )

smax=summary(model)$coeff[1,1]

tmin=summary(model)$coeff[2,1]

tmax=summary(model)$coeff[3,1]

alpha=summary(model)$coeff[4,1]

beta=summary(model)$coeff[5,1]

numberpoints=trunc((tmax-tmin)*100,0)

value=matrix(0,(numberpoints+2),1);t=matrix(NA,(numberpoints+2),1)

for(i in 2:(numberpoints+1)){

t[i]=0.01*(i-1)+tmin

ratio=(t[i]-tmin)/{tmax-tmin}

value[i]=smax*(sin(pi*(ratio^alpha)))^beta

}

t[1]=tmin;t[numberpoints+2]=tmax

points(t,value,type='l',col='green')

allalpha[trial]=alpha

allbeta[trial]=beta

allsmax[trial]=smax

alltmin[trial]=tmin

alltmax[trial]=tmax

startt=finisht+1

finisht=startt+length(temp)-1

allslopeplus[startt:finisht]=slopeplus

allseplus[startt:finisht]=seplus

alltemp[startt:finisht]=temp

ALLWEIGHT[startt:finisht]=WEIGHT

print(c(temp1,temp2,len,startt,finisht))

resid= slopeplus-predict(model)

weightedrss[trial]=sum(resid*resid*WEIGHT)

}

########## FITTING ALL SECTIONS AT ONCE #####

allf=function(t,allsmax,alltmin,alltmax,alpha,beta){

result=c(1:120)

s=t;ngroup=6;nstart=c(1,19,38,59,79,100);nend=c(18,37,58,78,99,120)

for(j in 1:ngroup){

diff=max(alltmax[j]-alltmin[j],0.0001);

for(i in nstart[j]:nend[j]){

num=max((t[i]-alltmin[j]),0.0);

z= pi*(num/diff)^alpha;

s[i]=max(sin(z),0.0)

}

result[nstart[j]:nend[j]]= allsmax[j]*(s[nstart[j]:nend[j]]^beta)

}

return(result)

}

topslope=max(allslopeplus,na.rm=TRUE)

toptemp=max(alltemp,na.rm=TRUE)

bottomtemp=min(alltemp,na.rm=TRUE)

allmodel=nls(as.vector(allslopeplus)~allf(as.vector(alltemp),allsmax,alltmin,alltmax,alpha,beta),

weights=ALLWEIGHT,

algorithm='port',

trace=TRUE,

start=list(allsmax=c(rep((0.5*topslope),each=ngroup)),alltmin=c(rep((alltemp[1]+0.1),each=ngroup)),

alltmax=c(rep((toptemp-0.1),each=ngroup)),

alpha=1.0,beta=0.4),

lower=c(c(rep((0.005*topslope),each=ngroup)),c(rep(bottomtemp,each=ngroup)),

c(rep((toptemp-2),each=ngroup)),0.1,0.1),

upper=c(c(rep(topslope,each=ngroup)),c(rep((bottomtemp+2),each=ngroup)),

c(rep((toptemp),each=ngroup)),2,2))

summary(allmodel)

resid=allslopeplus-predict(allmodel)

sum(resid*resid*ALLWEIGHT)

allsmaxfit=c(1:6);allsmaxfit[1:6]=summary(allmodel)$coeff[1:6,1]

alltmaxfit=c(1:6);alltmaxfit[1:6]=summary(allmodel)$coeff[13:18,1]

alltminfit=c(1:6);alltminfit[1:6]=summary(allmodel)$coeff[7:12,1]

commonalpha=summary(allmodel)$coeff[19,1]

commonbeta=summary(allmodel)$coeff[20,1]

########## PLOTTING ALL SECTIONS AT ONCE ##########

ngroup=6;nstart=c(1,19,38,59,79,100);nend=c(18,37,58,78,99,120)

for (i in 1:ngroup){

plot(alltemp[nstart[i]:nend[i]],allslopeplus[nstart[i]:nend[i]],xlab="Temperature (degrees C)",

ylab="Growth rate (per day)",xlim=c(alltemp[100],alltemp[120]),ylim=c(0,0.40),main=title[i])

#for(j in 1:nid){lines(c(temps[j+temp1-1],temps[j+temp1-1]),c(slope[j]+se[j],slope[j]-se[j]),col="red")

allvalue=matrix(0,101,1)

step=(alltmaxfit[i]-alltminfit[i])/100

for(j in 1:99){

ratio=(step*j)/(alltmaxfit[i]-alltminfit[i])

allvalue[j+1]=allsmaxfit[i]*(sin(pi*(ratio^commonalpha)))^commonbeta

}

allvalue[1]=0;allvalue[101]=0

x=alltminfit[i]+c(0:100)*step

points(x ,allvalue,type='l',col='green')

#########################

x=c(alltmin[i],alltemp[(nstart[i]+1):(nend[i]-1)],alltmax[i])

y=c(0,value[(nstart[i]+1):(nend[i]-1)],0)

value=matrix(0,101,1)

step=(alltmax[i]-alltmin[i])/100

for(j in 1:99){

ratio=(step*j)/(alltmax[i]-alltmin[i])

value[j+1]=allsmax[i]*(sin(pi*(ratio^allalpha[i])))^allbeta[i]

}

value[1]=0;value[101]=0

x=alltmin[i]+c(0:100)*step

points(x ,value,type='l',col='red')

}
