# Supplementary material for: A Key Marine Diazotroph in a Changing Ocean: The Interacting Effects of Temperature, CO2 and Light on the Growth of Trichodesmium erythraeum IMS101
Source: PLoS One. 2017 Jan 12;12(1):e0168796. doi: 10.1371/journal.pone.0168796 (PMC5230749; doi:10.1371/journal.pone.0168796)
Supplement: S1 Table — Temperature response growth conditions; low (~ 180 ppm), mid (~ 380 ppm) and high (~ 720 ppm) CO2, 40 μmol photons m-2 s-1 (LL) and 400 μmol photons m-2 s-1 (HL), ranging between 18–31°C. Light response growth conditions; low (~ 180 ppm), mid (~ 380 ppm) and high (~ 720 ppm) CO2, 26°C, ranging between 20–1400 μmol photons m-2 s-1). A circle (O) represents a growing culture; a cross (X) represents a condition where growth did not occur; a dash (-) represents a condition that was not used for culturing. (DOCX) [file pone.0168796.s007.docx]

|  | **Temperature response** | | | | | |  | **Light response** | | | |
| --- | --- | --- | --- | --- | --- | --- | --- | --- | --- | --- | --- |
|  | **Low light** | | | **High light** | | |  |  |  |  |  |
| **Temperature (°C)** | **Low CO_2_** | **Mid CO_2_** | **High CO_2_** | **Low CO_2_** | **Mid CO_2_** | **High CO_2_** |  | **Light (µmol photons m^-2^ s^-1^)** | **Low CO_2_** | **Mid CO_2_** | **High CO_2_** |
| 19.0 | X | X | X | X | X | X |  | 1390 | O | O | - |
| 19.7 | X | O | O | X | X | O |  | 1378 | O | O | O |
| 20.3 | X | O | O | X | O | O |  | 1370 | O | O | - |
| 21.3 | O | O | O | O | O | O |  | 1330 | - | - | O |
| 21.8 | O | O | O | O | O | O |  | 1257 | - | O | - |
| 22.4 | O | O | O | O | O | O |  | 1185 | O | O | - |
| 22.9 | O | O | O | O | O | O |  | 1150 | - | O | - |
| 23.4 | O | O | O | O | O | O |  | 1130 | - | - | O |
| 23.9 | O | O | O | O | O | O |  | 1066 | O | O | - |
| 24.5 | O | O | O | O | O | O |  | 1017 | O | O | - |
| 25.0 | O | O | O | O | O | O |  | 993 | O | O | - |
| 25.6 | O | O | O | O | O | O |  | 945 | O | O | O |
| 26.2 | O | O | O | O | O | O |  | 720 | O | O | O |
| 26.7 | O | O | O | O | O | O |  | 500 | O | O | O |
| 27.3 | O | O | O | O | O | O |  | 400 | O | O | O |
| 27.9 | O | O | O | O | O | O |  | 320 | O | O | O |
| 28.5 | O | O | O | O | O | O |  | 240 | O | O | O |
| 29.2 | O | O | O | O | O | O |  | 160 | O | O | O |
| 29.9 | O | O | O | O | O | O |  | 80 | O | O | O |
| 30.5 | X | O | O | O | O | O |  | 40 | O | O | O |
| 31.1 | X | X | X | X | X | X |  | 20 | O | O | O |
|  |  |  |  |  |  |  |  | 10 | X | X | X |
