# Supplementary material for: A Key Marine Diazotroph in a Changing Ocean: The Interacting Effects of Temperature, CO2 and Light on the Growth of Trichodesmium erythraeum IMS101
Source: PLoS One. 2017 Jan 12;12(1):e0168796. doi: 10.1371/journal.pone.0168796 (PMC5230749; doi:10.1371/journal.pone.0168796)
Supplement: S2 Table — Footnotes to S2 Table. a CO2 fixation to carbohydrate in the Calvin cycle according to the following stoichiometry. CO2 + 3 ATP + 2 NADPH → CH2O + H2O + 3ADP +3 Pi. The photon requirement (9 photons/CO2 fixed) is from Raven et al. [84]. b Carbon concentrating mechanism where the only energised step is the influx of HCO3- at one membrane between the medium and Rubisco. Lower value assumes no leakage, whereas the upper value assumes leakage rate equals to the rate of photosynthesis [84]. c The energetic cost of N2 fixation was calculated assuming complete recycling of H2 to recover ATP was calculated from the following stoichiometry: N2 + 6 H+ + 6 e- + 13 ATP → 2 NH3 + 13 ADP + 13 Pi.d The cost of ammonium assimilation into amino acids is 1 ATP/NH3 and 1 NADPH (2 reducing equivalents) assimilated via GOGAT. Protein synthesis would require an additional 4 ATP per peptide bond formed. e Based on a typical photosynthetic quotient of 1.2 O2 evolved per CO2 fixed for algae growing with ammonium as the inorganic N source. This accounts for the more reduced state of lipids and proteins relative to carbohydrates. f Total cost of synthesising 1 unit of C-biomass assumes a Redfield C:N ratio of 106C:16N and that protein accounts for all of the cell N. g Photon requirements were calculated based on 1/3 ATP generated per photon absorbed during linear photosynthetic electron transfer from H2O to O2, with the additional ATP requirement from provided either by LPET from H2O to H2O (water-water cycle) with 1/3 ATP generated per photon absorbed (higher estimate) or by cyclic photosynthetic electron transfer around photosystem I with 1 ATP generated per photon absorbed. (DOCX) [file pone.0168796.s008.docx]

| **Process** | **Reducing equivalent requirement** | **ATP requirement** | **Photon requirement** |
| --- | --- | --- | --- |
| CO_2_ fixation via Calvin Cycle^a^ | 4 e^-^/CO_2_ fixed | 3 ATP/CO_2_ fixed | 9 photons/CO_2_ fixed |
| Cyanobacterial CCM^b^ | - | - | 0.5-2 photons/CO_2_ fixed |
| N_2_ fixation^c^ | 3 e^-^/N fixed in NH_3_ | 6.5 ATP/N fixed | 11-19.5 photons/N fixed |
| N assimilation^d^ | 2 e^-^/NH_4_^+^ assimilated into protein | 5 ATP/NH_4_^+^ assimilated into protein | 8-15 photons/NH_4_^+^ assimilated into protein |
| Reduction of CH_2_O to C-biomass^e^ | 0.8/CO_2_ fixed | - | 1.8 photons/C-biomass produced |
| Total cost of producing 1 unit of C-biomass^f^ | 5.55 e-/C | 4.7 ATP/C-biomass produced | 14-16 photons/C-biomass produced |
