# Supplementary material for: A Key Marine Diazotroph in a Changing Ocean: The Interacting Effects of Temperature, CO2 and Light on the Growth of Trichodesmium erythraeum IMS101
Source: PLoS One. 2017 Jan 12;12(1):e0168796. doi: 10.1371/journal.pone.0168796 (PMC5230749; doi:10.1371/journal.pone.0168796)
Supplement: S3 Table — Key; x is the calculated value, σx is the calculated error of uncertainty; a, b and c are known quantities; σa, σb and σc are errors of uncertainty for a, b and c, respectively; y is a constant with no measure of uncertainty. (DOCX) [file pone.0168796.s009.docx]

| **Type** | **Example** | **Solution** |
| --- | --- | --- |

| Addition or Subtraction | $x =a+b-c$ | $\sigma_{x}=\sqrt{{\sigma_{a}}^{2}+{\sigma_{b}}^{2}+{\sigma_{c}}^{2}}$ |
| --- | --- | --- |
| Multiplication or Division | $x=\frac{a\cdot b}{c}$ | $\sigma_{x}=x\cdot\sqrt{\left( \frac{\sigma_{a}}{a} \right)^{2}+\left( \frac{\sigma_{b}}{b} \right)^{2}+\left( \frac{\sigma_{c}}{c} \right)^{2}}$ |
| Exponentiation | $x=a^{y}$ | $\sigma_{x}=y\cdot x\frac{\sigma_{a}}{a}$ |
| Natural Logarithm | $x=\ln a$ | $\sigma_{x}=\frac{\sigma_{a}}{a}$ |
